# Heart Rate Variability for the Early Detection of Cardiac Autonomic Dysfunction in Type 1 Diabetes

**DOI:** 10.3389/fphys.2022.937701

**Published:** 2022-06-30

**Authors:** Paolo Castiglioni, Andrea Faini, Anika Nys, Renee De Busser, Martijn Scherrenberg, Esmee Baldussu, Gianfranco Parati, Paul Dendale

**Affiliations:** ^1^ IRCCS Fondazione Don Carlo Gnocchi Onlus, Milan, Italy; ^2^ Istituto Auxologico Italiano, IRCCS, Milan, Italy; ^3^ UHasselt, Faculty of Medicine and Life Sciences, Diepenbeek, Belgium; ^4^ KU Leuven, Faculty of Medicine and Life Sciences, Leuven, Belgium; ^5^ Heart Centre Hasselt, Jessa Hospital, Hasselt, Belgium; ^6^ Faculty of Medicine and Health Sciences, Antwerp University, Belgium; ^7^ Maastricht UMC+, Maastricht, Netherlands; ^8^ Department of Medicine and Surgery, University of Milano-Bicocca, Milan, Italy

**Keywords:** heart rate variability, autonomic neuropathy, complexity, frequency-domain, entropy, self-similarity

## Abstract

Type 1 diabetes mellitus (T1DM) has an important impact on morbidity and mortality because it may start early in life. Therefore, the early detection of cardiovascular autonomic neuropathy (DCAN) in T1DM patients is important to intervene quickly and prevent further deterioration. Traditional autonomic function tests detect abnormalities in severely symptomatic patients but they are difficult to be standardized, require the patient’s active participation and their sensitivity to the early disease is limited. In comparison, heart rate variability (HRV) is easier to be measured and standardized. Therefore, we aim to find the HRV indexes that better identify DCAN at an early stage in T1DM patients, and evaluate if HRV is a valid alternative to traditional tests. For this aim, we administered the SCOPA-AUT questionnaire on symptoms of autonomic dysfunction as well as deep breathing, Valsalva, handgrip, head-up tilt (HUT), and cold-pressor tests, to 52 T1DM patients and 27 controls. We calculated HRV indexes during supine rest (SUP) and HUT, assessing differences between groups and postures by a linear mixed-effect model for repeated measures. Receiver Operating Characteristic (ROC) analysis quantified how each HRV index and autonomic test distinguishes between patients and controls. We found that the SCOPA-AUT score was slightly but significantly (*p* < 0.05) greater in patients, indicating an early DCAN. T1DM patients preserved the HRV response to changing posture but in SUP they showed significantly lower standard deviation and vagal indexes of HRV than controls. The area under the ROC curve of these HRV indexes was not lower than 0.68. By contrast, traditional autonomic tests did not differ between groups. Therefore, early DCAN initially causes an impairment of the cardiac vagal control manifest in conditions of elevated vagal tone, as in SUP. Compensatory adjustments of the sympathetic control might explain the unaltered response to traditional autonomic tests. In conclusion, vagal HRV indexes in SUP help to identify early DCAN better than traditional tests, potentially allowing rapid interventions.

## Introduction

Type 1 diabetes is a chronic disease that cannot be cured easily and that has an important impact on morbidity and mortality because it may start early in life. An often undiagnosed complication is autonomic neuropathy, which affects many organs, reduces the quality of life, and increases hospitalizations, healthcare costs, cardiovascular events, and mortality (Vinik et al., 2013). Therefore, the early detection of autonomic neuropathy in diabetic patients is important to intervene quickly and prevent further deterioration, e.g., by administering programs of exercise training (Bhati, Shenoy and Hussain, 2018).

The autonomic neuropathy induced by diabetes may lead to an altered autonomic control of circulation, or Diabetic Cardiovascular Autonomic Neuropathy (DCAN). DCAN prevalence reaches up to 35% in type 1 patients with long-standing diabetes (Spallone et al., 2011). The traditional method to detect DCAN consists of a battery of autonomic tests that investigate the integrity of the autonomic function eliciting cardiac or vascular responses (Ewing et al., 1985). These tests require the active participation of the subject and are not easy to be standardized. By contrast, heart rate variability (HRV) indexes can be easily obtained in controlled conditions without the patients’ active participation. Since HRV reflects the functioning of the autonomic nervous system (Karemaker, 2017), there is a rising interest in HRV to quantify the severity of DCAN (Benichou et al., 2018). Most of the literature on this issue regards the association between HRV and type-2 diabetes while little is known about the HRV changes in type-1 diabetes. Furthermore, whether HRV indexes are sensitive enough to detect DCAN at an early stage is still unknown.

Therefore, our main aim is to evaluate if HRV detects DCAN at an early stage in type-1 diabetic patients. In particular, we want to investigate what are the more relevant indexes among those used in the HRV literature and the more informative conditions for measuring these indexes. The secondary aim is to compare the sensitivity of these indexes in detecting early DCAN with the sensitivity of the traditional autonomic function tests.

## Methods

### Subjects

We considered a convenience sample with a diabetes/control ratio of around 2:1. We enrolled 52 subjects with type-1 diabetes mellitus (T1DM), contacted by the Endocrinology Department of a tertiary referral hospital. The initial diagnosis of diabetes was based on clinical symptoms and glycated hemoglobin test. The assessment of C-peptide and T1DM specific antibodies was performed to determine the diagnosis of T1DM. All patients had been under strict supervision from endocrinologists since their diagnosis and were not hospitalized during the last 3 months. All were taking rapid action insulin, administered by a pump in 24.5% of the cases and integrated by long-acting insulin in the remaining 75.5% of the cases. For inclusion, they had to be > 18 years with at least 2 years of diabetes. Exclusion criteria were uncontrolled hypertension (> 180/100 mmHg), atrial fibrillation or other cardiovascular diseases, limiting lung or orthopedic diseases, use of *β*- and α-blockers, antidepressants, and (anti)cholinergic medications. These enrollment criteria reasonably exclude the occurrence of HRV impairments by causes different from DCAN. We enrolled 27 controls (CNTR) selected among members of our hospital team and their families, matched by gender and age. All the participants gave written informed consent. The study was approved by the ethics committees of the recruiting center (Jessa Hospital Hasselt) and registered on ClinicalTrials.gov as NCT03481374.

### Experimental Protocol

Data were collected between September 2016 and March 2017 in a quiet environment with the Task-Force Monitor system (CNSystems Medizintechnik GmbH, Graz, Austria), which noninvasively measures beat-to-beat systolic and diastolic blood pressure at the finger artery and two ECG leads at 1,000 Hz. Recordings started with 16 min of supine rest (SUP) followed by the execution of 5 autonomic tests. These were the cold pressor test and the Ewing’s battery of autonomic tests: deep breathing, Valsalva maneuver, sustained handgrip, lying-to-standing. The first 4 tests, executed in a lying position and randomized sequence, lasted about 18 min. The last test was the head-up tilt (HUT), to be maintained for 10 min but shortened in case of signs of pre-syncope. The recording session ended a couple of minutes after the tilt table returned to the horizontal position.

### SCOPA-AUT Test

The SCOPA-AUT, a reliable and validated questionnaire evaluating the symptoms of autonomic dysfunction perceived in daily life (Pavy-Le Traon et al., 2011), was administered to patients and controls. It consists of 23 questions assessing gastrointestinal, urinary, cardiovascular, thermoregulatory, pupillomotor, and sexual dysfunctions. The final score is expressed as a percentage of the maximum achievable score between 0% and 100%.

### Autonomic Function Tests and Ewing’s Score

The deep breathing test was performed as in (Jyotsna et al., 2009): the difference between the maximum and minimum heart rate during deep breathing (Deep Breathing ΔHR) was taken as the autonomic function measure. The Valsalva test consisted of three Valsalva maneuvers (Jyotsna et al., 2009): the largest ratio among the three maneuvers between the longest RR interval in phase IV and the shortest RR interval in phase II (Valsalva ratio) was the outcome of the test. The sustained handgrip test consisted of holding a dynamometer at 30% of maximal voluntary contraction for 3 minutes: the outcome was the difference between the maximal diastolic blood pressure during the contraction and its baseline value (Handgrip ΔDBP). For the head-up tilt test, the tilt table was raised to 70° using leg straps to minimize the contribution of the muscle pump: outcomes were the ratio between the longest RR interval around the 30th beat and the shortest RR interval around the 15th beat (HUT 30:15 ratio) and the difference between the lowest systolic blood pressure (SBP) during the first 3 minutes of HUT and its baseline value (HUT ΔSBP). Ewing’s method gave a score equal to 0, 1/2, or 1 for a normal, borderline, or abnormal result in each test: see thresholds defining borderline and abnormal values in (Ewing et al., 1985). The final score may range between 0 and 5.

The cold pressor test consisted in maintaining the arm immersed up to the wrist in water at 4°C for 90 s. Outcomes were the differences between the maximal diastolic blood pressure during immersion and its baseline value (Cold pressor ΔDBP) and the difference between the highest heart rate during immersion and its baseline value (Cold pressor ΔHR).

### Heart Rate Variability Analysis

HRV indexes were calculated in SUP and HUT separately, having SUP a predominant parasympathetic cardiac control and HUT a substantial sympathetic cardiovascular activation and/or vagal deactivation. We applied a derivative-and-threshold algorithm to the ECG to identify the position of the R peaks, refining their position with parabolic interpolation (Task Force of the European Society of Cardiology and The North American Society of Pacing Electrophysiology, 1996). An interactive procedure removed premature beats and artifacts and only cardiac intervals from normal sinus node depolarization (normal-to-normal, or NN, intervals) were considered. The average NN interval, NNI_m_, was calculated in SUP and HUT.

We considered HRV indexes in the time, frequency, and complexity domains. In the time domain, we measured pNN50+, the percentage of NN intervals at least 50-ms longer than their preceding NN interval (Ewing, Neilson and Travis, 1984); pNN50-, the percentage of NN intervals at least 50-ms shorter than their preceding NN interval; and RMSSD, root-mean-square of successive NN differences, all indexes of cardiac vagal activity. We calculated the SDNN-index (SDNN_i_) as the mean of the standard deviations of NN intervals over a running data window of 5 min (Task Force of the European Society of Cardiology and The North American Society of Pacing Electrophysiology, 1996).

Before the frequency-domain analysis, we resampled the beat-by-beat NN intervals at 5 Hz, linearly interpolating possibly missing beats. We calculated the Welch periodogram with 50%-overlapped Hann data windows of 120-s length and wideband spectral smoothing (Di Rienzo et al., 1996). Powers in the very-low-frequency (VLF, between 0.005 and 0.04 Hz), low-frequency (LF, between 0.04 and 0.15 Hz), and high-frequency (HF, between 0.15 and 0.40 Hz) bands, total power (TOT_P_, between 0 and 0.5 Hz) and the LF/HF powers ratio were obtained integrating the periodogram (Task Force of the European Society of Cardiology and The North American Society of Pacing Electrophysiology, 1996).

In the complexity domain, we calculated HRV entropy (a measure of irregularity) and self-similarity (a measure of fractal dimension). We estimated sample entropy, SampEn (Richman and Moorman, 2000), setting the embedding dimension *m =* 1 and the tolerance *r =* 15% the standard deviation of the series. We calculated the multiscale entropy, MSE, which quantifies SampEn as a function of the time scale *τ*, MSE(*τ*) (Costa, Goldberger and Peng, 2002), setting *m* = 1 and a fixed-tolerance *r* = 15% the standard deviation (Castiglioni et al., 2017). We extracted two entropy indexes, MSE_HF_ and MSE_LF_, averaging MSE(*τ*) over the scales corresponding to the HF (i.e., 2.5 ≤ τ < 6.7 s) and LF (6.7 ≤ τ < 25 s) bands (Castiglioni, Parati and Faini, 2019). As a self-similarity estimator, we considered the multiscale detrended fluctuation analysis, α(*τ*) (Castiglioni et al., 2011). We extracted two indexes from α(*τ*): the short-term coefficient, α_Short_, averaging α(*τ*) between *τ* = 5 and *τ* = 11 s; and the long-term coefficient, α_Long_, averaging α(*τ*) over 17 < τ < 61 s.

### ECG-Derived Respiration

A respiratory signal was extracted from the fluctuations of QRS-complex amplitude of the ECG reflecting the respiratory movements of the thorax (Moody et al., 1986). The series of R peaks were sampled at 5 Hz and high-pass filtered at 0.05 Hz to remove oscillations too long to be generated by respiratory movements. The breathing rate was the frequency of the highest spectral peak of the periodogram.

### Baroreflex Function

We resampled the beat-by-beat SBP series at 5 Hz and calculated the baroreflex sensitivity on the heart rate with the transfer function method over the LF band (Robbe et al., 1987). This is based on the hypothesis that a resonance frequency in the baroreflex loop produces blood pressure oscillations in the LF band due to the time delay in the response of the vascular smooth muscles and that the baroreflex responds to such oscillations modulating the heart rate at the same frequency. The transfer function between blood pressure and heart rate was calculated by the Welch periodogram as the ratio between the SBP-NNI cross-spectrum modulus and the root-squared SBP spectrum, in ms/mmHg, and averaged over the LF band. The SBP-NNI coherency spectrum over the LF band was calculated as an index of the baroreflex-mediated coupling between blood pressure and heart rate.

### Statistics

Preliminary power analysis demonstrated that the sample size is adequate to detect a 15% alteration of SDNN_i_ (index of global HRV) considering an expected value of 62.2 (13) ms, as tabulated in (Umetani et al., 1998) for healthy subjects around 40 yoa (two-tailed nonparametric test with alpha = 0.05 and power = 80%). We compared the general characteristics, SCOPA-AUT scores, and outcomes of the autonomic tests between patients and controls by the Mann-Whitney U test (ordinal data) or the Fisher’s exact test (categorical data). HRV indexes were compared between groups and postures by a Linear Mixed-Effects Model that provides the statistical significance of the factors Group (CNTR vs. T1DM) and Condition (SUP vs. HUT) and their interaction. Gaussianity of the residuals was tested; if the test failed, the data were log- or rank-transformed. When one of the factors or their interaction was significant at *p* < 0.05, we tested the differences between SUP and HUT for each group and the differences between patients and controls in each condition with *a posteriori* contrasts, accounting for multiple comparisons with the false discovery rate correction. All tests were two-tailed at *p* < 0.05. Receiver Operating Characteristic (ROC) analysis was performed for the HRV indexes and autonomic tests. The Area Under the ROC Curve (AUC) measured how the index performs as a classifier between cases and controls. When the AUC was significant at *p* < 0.05, the Youden index indicated the cut-off value for the classification (Goksuluk et al., 2016). Statistical analysis was performed with “R: A language and environment for statistical computing. R Foundation for Statistical Computing,” R Core Team (2019).

## Results

At the time of data collection, it turned out that four patients were taking a *β*-blocker not registered in the hospital medical files and were excluded. The ECG quality was too low for the HRV analysis in two controls and the HUT test had to be interrupted after 3 min in another control. Therefore, the HRV analysis was conducted on 48 patients, 25 controls in SUP, and 24 controls in HUT. Individual values of glycated hemoglobin and years since the T1DM diagnosis in the patients’ group are reported in the Supplemental Figure S1.

The two groups were matched in terms of age, gender ratio, and anthropometric measures (Table 1). The perceived level of autonomic dysfunction quantified by the SCOPA-AUT questionnaire was slightly but significantly greater in the patients’ group while Ewing’s scores and the autonomic tests were similar in patients and controls (Table 1).

**TABLE 1:** General characteristics and outcomes of the autonomic function tests in T1DM and CNTR groups: mean (SD) or median [IQR] with statistical significance of the difference between groups.

	T1DM (N=48)	CNTR (N=25)	*p*-value
*General Characteristics*			
Age (yoa)	35.0 (13.0)	35.6 (12.9)	0.71
women/men ratio	19/29	12/13	0.62
height (cm)	174.3 (9)	172.9 (10.1)	0.54
weight (kg)	74.6 (13.6)	73.5 (16.8)	0.62
body mass index (kg/m^2^)	24.5 (3.9)	24.3 (3.7)	0.93
Years since T1DM diagnosis	15.4 (18.7)	—	—
Glycated hemoglobin (%)	7.55 (1.32)	—	—
*SCOPA-AUT Questionnaire*			
Score (%)	10.9% [11.5%]	7.2% [5.4%]	0.018
*Ewing Battery of Autonomic Tests*			
Deep Breathing ΔHR (bpm)	17.3 (7.4)	20.2 (8.5)	0.09
Valsalva ratio	1.77 (0.43)	1.75 (0.31)	0.79
Handgrip ΔDBP (mmHg)	21.9 (11.2)	20.2 (11.4)	0.42
HUT 30:15 ratio	1.34 (0.18)	1.44 (0.21)	0.11
HUT ΔSBP (mmHg)	−9.4 (11.9)	−8.0 (11.2)	0.29
Total Ewing score	0.66 (0.72)	0.57 (0.56)	0.68
*Cold Pressor Test*			
ΔDBP (mmHg)	18.5 (11.3)	15.5 (10.9)	0.22
ΔHR (bpm)	15.2 (6.9)	15.1 (7.6)	0.83

*p* after Fisher's exact test for gender composition, after Mann Whitney U test for the other variables.

### Heart Rate Variability Analysis

**FIGURE 1:**
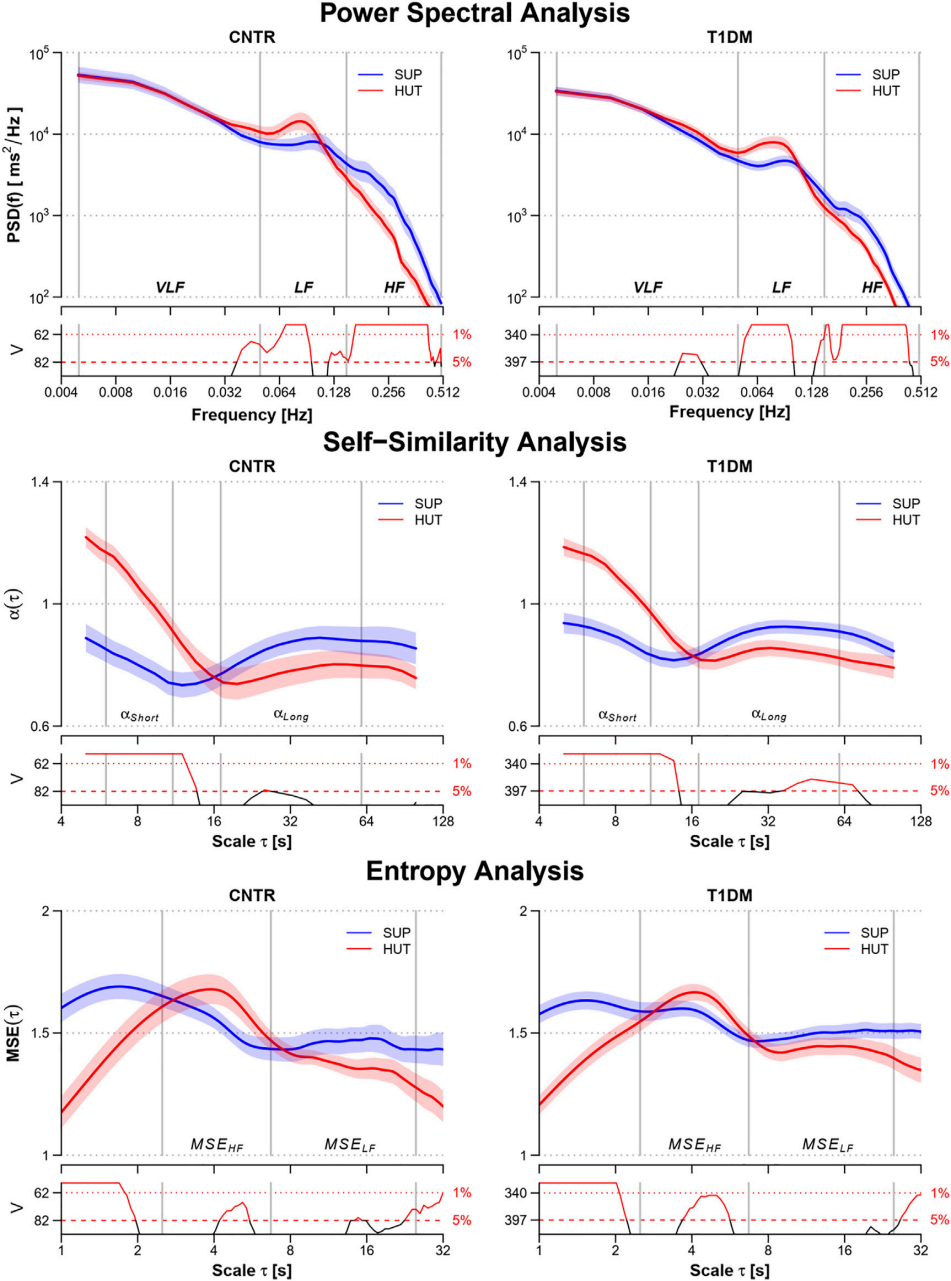
Power spectra, PSD(f), multiscale self-similarity coefficients, α(τ), and multiscale entropy, MSE(τ), in the control (CNTR) group and type-1 diabetic (T1DM) group during supine rest (SUP) and head-up tilt (HUT). From top to bottom: geometric mean ± geometric sem for PSD(f), mean ± sem for α(τ) and MSE(τ). Grey vertical lines delimit the VLF, LF, and HF frequency bands of the power spectra; the short- and long-scale ranges of the self-similarity coefficients; and the HF and LF components of the multiscale entropy. The value of the V statistics of the Mann-Whitney test is reported below each panel: when the V value is above the dashed (dotted) horizontal lines, the difference between the SUP and HUT postures at the corresponding frequency or scale is statistically significant at the 5% (1%) significance level.

The shift of the cardiac autonomic control from a predominant vagal tone in SUP to the sympathetic activation in HUT appears clearly in power spectra, self-similarity profiles, and multiscale entropies of both groups (Figure 1). It produced a higher LF spectral peak and lower HF power, greater short-term self-similarity, and lower entropy at the shortest scales.

The factors Group and Condition were significant for NNI_m_ that was lower in the T1DM group during SUP and that decreased in both groups from SUP to HUT, reaching similar values (Table 2 and Supplemental Figure S2). Group and Condition were significant also for the time-domain indexes pNN50+, pNN50- and RMSSD (Table 2), lower in the T1DM group during SUP and which decreased from SUP to HUT reaching similar values in patients and controls. The significant Group × Condition interaction for pNN50+ highlights its greater decrease from SUP to HUT in controls. Only the factor Group was significant for SDNN_i_ and the patients had lower SDNN_i_ both in SUP and HUT (Supplemental Figure S3).

**TABLE 2:** NNI_m_ and HRV Indexes by groups and conditions: mean (SD) with statistical significance of the factors after linear mixed-effects model.

		SUP	HUT	*p*-value		
				Group	Condition	Group × Condition
NNI_m_ (ms)	CNTR	922 (147)	737 (104)^b^	**0.022**	**<0.001**	0.1
	T1DM	858 (104)^a^	711 (99)^b^			
*Time Domain*						
pNN50+ (%)	CNTR	11.2 (9.2)	4.2 (4.7)^b^	**0.008**	**<0.001**	**0.046**
	T1DM	6.6 (7.3)^a^	2.7 (3.2)^b^			
pNN50- (%)	CNTR	11.8 (9.8)	4.0 (4.6)^b^	**0.005**	**<0.001**	0.2
	T1DM	7.1 (8.7)^a^	2.2 (3.3)^b^			
RMSSD (ms)	CNTR	50.5 (31.5)	28.5 (15.3)^b^	**0.002**	**<0.001**	0.1
	T1DM	33.1(21.5)^a^	21.6 (10.0)^b^			
SDNN_i_	CNTR	62.1 (27.8)	58.0 (20.2)	**0.002**	0.7	0.5
	T1DM	44.4 (18.0)^a^	45.9 (16.2)^a^			
*Frequency Domain*						
TOT_P_ (ms^2^)	CNTR	4591 (4180)	3700 (2787)	**0.002**	0.9	0.5
	T1DM	2261 (1701)^a^	2270 (1459)^a^			
VLF (ms^2^)	CNTR	1555 (1482)	1188 (517.4)	**0.011**	0.9	0.8
	T1DM	856 (547)^a^	895 (536.9)^a^			
LF (ms^2^)	CNTR	1258 (1149)	1793 (2168)	**0.012**	0.07	0.9
	T1DM	672 (603.3)^a^	927 (809.9)^a^			
HF (ms^2^)	CNTR	1087 (1741)	336 (331)^b^	**0.004**	**<0.001**	0.1
	T1DM	459 (633)^a^	201 (200)^b^			
LF/HF	CNTR	2.03 (1.56)	6.58 (4.24)^b^	0.3	**<0.001**	0.2
	T1DM	2.45 (1.66)	6.48 (5.02)^b^			
*Complexity Domain*						
α_SHORT_	CNTR	0.817 (0.187)	1.089 (0.159)^b^	0.06	**<0.001**	0.1
	T1DM	0.898 (0.187)	1.108 (0.146)^b^			
α_LONG_	CNTR	0.856 (0.175)	0.776 (0.218)	0.3	0.06	0.8
	T1DM	0.903 (0.140)	0.836 (0.167)			
SampEn	CNTR	1.551 (0.332)	1.009 (0.323)^b^	>0.9	**<0.001**	0.5
	T1DM	1.520 (0.293)	1.045 (0.336)^b^			
MSE_HF_	CNTR	1.540 (0.194)	1.622 (0.264)^b^	>0.9	**0.007**	0.4
	T1DM	1.562 (0.204)	1.609 (0.227)			
MSE_LF_	CNTR	1.455 (0.254)	1.373 (0.162)	0.2	**0.044**	0.7
	T1DM	1.494 (0.188)	1.437 (0.232)			

*p*-values of significant (p<0.05) factors in bold.

a indicates a significant difference between CNTR and T1DM in a given condition.

b significant difference between SUP and HUT in a given group, after false discovery rate correction for multiple comparison.

In the frequency domain, the factor Group was significant for the spectral powers, lower in patients during SUP, but not for the LF/HF powers ratio (Table 2 and Supplemental Figure S4). The factor Condition was significant for the LF/HF powers ratio, which increased from SUP to HUT without differences between groups, and for the HF power, which decreased from SUP to HUT reaching similar values in patients and controls. The factor Group was significant also for TOT_p_, with lower total spectral power in the T1DM group both in SUP (*p* <1%) and HUT (*p* <5%).

The respiratory frequency was equal to 0.18 (0.09) Hz during SUP and 0.15 (0.08) Hz during HUT in controls, equal to 0.20 (0.09) Hz during SUP and 0.19 (0.09) Hz during HUT in patients: neither the Group (*p* = 0.07) nor the Condition (*p* = 0.06) or their interaction (*p* = 0.50) were significant. The change of posture increased α_Short_ and decreased SampEn but complexity indexes did not differ between groups (Table 2 and Supplemental Figure S5).

The baroreflex sensitivity decreased from SUP to HUT both in controls, from 13.97 (8.08) to 8.66 (3.95) ms/mmHg, and in patients, from 11.65 (7.09) to 6.80 (4.21) ms/mmHg, being significant the Condition factor (*p* < 10^−3^) but not the Group factor (*p* = 0.11) or their interaction (*p* = 0.50). The coherency spectrum over the LF band increased from SUP to HUT in controls, from 0.40 (0.17) to 0.52 (0.12), and patients, from 0.46 (0.17) to 0.54 (0.17). Also in this case, only the Condition factor was significant (*p* < 10^−3^), not the Group factor (*p* = 0.28) or their interaction (*p* = 0.39).

### Receiver Operator Characteristic Analysis

The score of the SCOPA-AUT questionnaire was associated with a significant area under the ROC curve, high specificity but a relatively low sensitivity (Table 3). By contrast, the AUC was not significant for each traditional autonomic test (Table 3). The AUC was also not significant for Ewing’s score, being AUC (SE) = 0.51 (0.07) with *p* = 0.93. On the other hand, the AUC was significant in SUP for all the HRV time-domain indexes and the total, LF, and HF powers. In particular, SDNN_i_ reached AUC = 0.70 (a value higher than the AUC of the SCOPA-AUT questionnaire) and the SDNN_i_<57 ms cut-off classifies participants in the T1DM group with 75% sensitivity and 60% specificity. Time-domain vagal indexes (pNN50, RMSSD) had higher specificity than sensitivity while frequency-domain indexes had higher sensitivity than specificity. Most AUCs were lower in HUT where only SDNN_i_, TOT_P,_ and the VLF power had significant AUCs, with high specificity (> 90%) but low sensitivity (≤ 40%).

**TABLE 3:** Area under the ROC curve (AUC) for the traditional autonomic tests and for HRV indexes by condition, with significance p, cut-off value, sensitivity and specificity for patients’ identification.

	**SUP**				**HUT**			
	AUC (SE)	*p*	Cut-off	Sn; Sp	AUC (SE)	*p*	Cut-off	Sn; Sp
*Scopa AUT Questionnaire^a^*								
Score%	0.67 (0.06)	0.007	>13%	40%; 92%	—	—	—	—
*Traditional Autonomic Tests*								
Deep Breath ΔHR	0.62 (0.07)	0.10	—	—	—	—	—	—
Handgrip ΔDBP	0.56 (0.07)	0.41	—	—	—	—	—	—
Valsalva ratio	0.52 (0.07)	0.77	—	—	—	—	—	—
Cold Pressor ΔDBP	0.52 (0.08)	0.84	—	—	—	—	—	—
Cold Pressor ΔHR	0.59 (0.07)	0.23	—	—	—	—	—	—
HUT 30:15 ratio	—	—	—	—	0.62 (0.07)	0.08	—	—
HUT ΔSBP	—	—	—	—	0.58 (0.07)	0.28	—	—
*HRV Indexes*								
NNI_m_	0.63 (0.07)	0.09	—	—	0.56 (0.07)	0.44	—	—
pNN50+	0.68 (0.06)	0.006	<4.3 %	56%; 72%	0.57 (0.08)	0.36	—	—
pNN50-	0.68 (0.06)	0.005	<2.1 %	44%; 92%	0.63 (0.07)	0.07	—	—
RMSSD	0.69 (0.07)	0.004	<26 ms	48%; 84%	0.62 (0.07)	0.10	—	—
SDNN_i_	0.70 (0.07)	0.003	<57 ms	75%; 60%	0.67 (0.07)	0.012	<40 ms	35%; 92%
TOT_P_	0.69 (0.07)	0.005	<3329 ms^2^	81%; 56%	0.68 (0.06)	0.005	<1603 ms^2^	40%; 96%
VLF	0.64 (0.07)	0.05	<1438 ms^2^	87.5%; 36%	0.65 (0.07)	0.02	<460 ms^2^	29%; 96%
LF	0.67 (0.07)	0.01	<1250 ms^2^	87.5%; 40%	0.63 (0.07)	0.08	—	—
HF	0.68 (0.07)	0.009	<686 ms^2^	81%; 52%	0.63 (0.07)	0.06	—	—
LF/HF	0.58 (0.07)	0.28	—	—	0.53 (0.07)	0.69	—	—
α_SHORT_	0.61 (0.07)	0.11	—	—	0.53 (0.07)	0.70	—	—
α_LONG_	0.58 (0.07)	0.25	—	—	0.59 (0.08)	0.22	—	—
SampEn	0.52 (0.08)	0.82	—	—	0.53 (0.07)	0.67	—	—
MSE_HF_	0.54 (0.07)	0.59	—	—	0.54 (0.08)	0.63	—	—
MSE_LF_	0.54 (0.08)	0.59	—	—	0.59 (0.07)	0.18	—	—

a Administered in sitting position; Sn, Sensitivity; Sp, Specificity; Cut-off values by the Youden method only for significant AUC.

## Discussion

There are three novel findings in our study. First, HRV indexes from a few minutes only of heart rate recordings can detect DCAN in type-1 diabetes even at such an early stage to be undetectable by traditional autonomic tests. Second, DCAN early alterations are clearer in SUP than in HUT. Third, specificity and sensitivity to predict DCAN depend on the family of HRV indexes (time-, frequency- or complexity-domain indexes) and the posture during the measure. Our results may have clinical relevance, possibly facilitating the early detection of DCAN in type-1 diabetes mellitus and monitoring of its progression over time. The following paragraphs discuss these points more in detail.

### Supine Rest vs. HUT

We considered postures with different autonomic activations: large vagal and small sympathetic modulations of heart rate in SUP and a marked sympathetic activation with vagal deactivation in HUT. Alterations affecting the autonomic branch forced to work at an elevated outflow are likely to be amplified and more easily detected. We showed that indexes of vagal modulations of heart rate (pNN50, RMSSD, and HF power) and sympathovagal balance (LF/HF, α_SHORT_, SampEn) follow the expected shift from a mainly vagal to a mainly cardiac sympathetic control from SUP to HUT (Task Force of the European Society of Cardiology and The North American Society of Pacing Electrophysiology, 1996; Castiglioni et al., 2007, 2011; Porta et al., 2013) in both groups (Figure 1). Among the indexes with a significant “Condition” factor (i.e., where the change of posture produced a significant change) only pNN50+ interacted with the “Group” factor. Thus the change of posture produced similar HRV changes in patients and controls, except for pNN50+ which, however, decreased from SUP to HUT in both groups too, its significant interaction highlighting a more pronounced reduction in controls. Therefore, DCAN did not prevent the physiological postural modulations and our T1DM patients responded with HRV changes in a physiologically similar way to controls.

### Detection of Cardiovascular Autonomic Neuropathy and Vagal Heart Rate Variability Indexes

Interestingly, all the markers of vagal heart rate modulations, i.e., pNN50+ and pNN50−, RMSSD, and HF power, were characterized by a highly significant “Group” factor (*p* < 0.01). The difference consisted of lower vagal indexes for the diabetic patients but only in SUP. This suggests that in our patients DCAN produced an initial impairment of the cardiac vagal control that becomes manifest in conditions of elevated vagal tone, like supine rest. The ROC analysis (Table 3) demonstrated that all the vagal HRV indexes might identify DCAN at an early stage, but only when measured in the SUP position.

### Detection of Cardiovascular Autonomic Neuropathy and Sympathovagal Heart Rate Variability Indexes

While no HRV indexes are pure markers of the cardiac sympathetic modulations of heart rate, the LF/HF powers ratio, the short-term self-similarity coefficient, and the sample entropy may be considered markers of the cardiac sympathovagal balance (Task Force of the European Society of Cardiology and The North American Society of Pacing Electrophysiology, 1996; Castiglioni et al., 2011; Porta et al., 2013). These indexes changed very significantly (*p* < 0.001) from SUP to HUT when we expected an increase in the sympathetic component and a reduction in the vagal component of the heart rate control. However, we did not find differences between groups in SUP or HUT. This suggests that DCAN did not prevent our T1DM patients to adjust their cardiac sympathetic control to compensate for the reduced vagal control and to preserve the physiological level of the sympathovagal balance.

### Detection of Cardiovascular Autonomic Neuropathy and Traditional Autonomic Tests

None of the traditional autonomic tests that quantify the DCAN severity revealed differences between patients and controls. This finding, however, is coherent with the similar HRV indexes of sympathovagal balance observed in the two groups and is understandable considering our hypothesis that adjustments of the cardiac sympathetic control compensate for impaired vagal control. The Valsalva, cold-pressor, and handgrip tests, even if performed in the supine position, elicit a sympathetic activation which may have masked the effects of the impaired vagal control. As to the deep-breathing test, the induced heart rate changes are not a pure marker of vagal modulations because they occur at 0.1 Hz where they are driven by both the vagal and sympathetic outflows, differently from the HF power which reflects respiratory oscillations modulated by the vagal outflow only (Task Force of the European Society of Cardiology and The North American Society of Pacing Electrophysiology, 1996). Thus, we may hypothesize that the traditional autonomic tests did not detect an initial cardiac vagal impairment in our patients because their outcomes reflect adjustments in the cardiac sympathetic response compensating for the partially impaired vagal response.

### Detection of Cardiovascular Autonomic Neuropathy and Other Time- and Frequency-Domain Heart Rate Variability Indexes

We found significant differences between groups also in indexes that are not pure vagal markers of heart rate modulations. One is the LF power, which reflects heart rate oscillations induced by both vagal and cardiac sympathetic modulations. Its lower value in patients during SUP and HUT can be explained by the occurrence of the previously mentioned concomitant factors: the impaired vagal response and the compensatory reduced sympathetic drive to preserve the physiological level of sympathovagal balance. The VLF power too is lower in patients. Although the renin-angiotensin-aldosterone system and the thermoregulatory system may play a role (Shaffer and Ginsberg, 2017), the major determinant of the VLF power is the parasympathetic nervous system (Taylor et al., 1998) and impaired vagal control may explain the reduced VLF power in the patients. The total power is almost equal to the sum of the VLF, LF, and HF powers, explaining why the Group factor is significant for TOT_P_, as for the VLF, LF, and HF powers separately. The standard deviation of NN intervals over a given data window is mathematically equal to the square root of the total power over the same window, explaining why SDNN_i_, like TOT_P_, was lower in patients. Interestingly, ROC analysis indicates that, unlike the pure vagal indexes, the indexes of global variability, TOT_P_ and SDNN_i_, classify between patients and controls also in HUT, even if with lower sensitivity and higher specificity than in SUP. This would suggest a certain degree of independence in the information on DCAN provided by global and purely vagal indexes of HRV.

In literature, a lower number of studies investigated the HRV alterations in type-1 than in type-2 diabetes. Their results, however, are coherent with our findings. Lower RMSSD, SDNN_i,_ and spectral powers without differences in the sympathovagal balance were found in type-1 diabetic children than in controls from 24-h Holter recordings (Kardelen et al., 2006), and in supine measures collected in pubertal type-1 diabetic girls (Cho et al., 2014) and young type-1 diabetic patients (Silva et al., 2017). In supine type-1 diabetic patients, RMSSD was lower and the LF/HF powers ratio was greater than in controls (Jaiswal et al., 2013) but only in the patients’ subgroup with the worse glycemic control, and in type-1 diabetic children, the impaired glucose control correlated inversely with spectral powers (Chen et al., 2007). These studies are thus consistent with our conclusion that DCAN at its early stage is associated with a vagal impairment and preserved sympathovagal balance.

### Detection of Cardiovascular Autonomic Neuropathy and Heart Rate Variability Complexity

Self-similarity differentiated the HRV dynamics between SUP and HUT but did not reveal differences between patients and controls and entropy indexes did not differ between groups even if they followed the postural shift in the sympathovagal balance. However, significantly lower values of entropy at scales of 2, 3, and 4 beats were reported in type-1 diabetes (Javorka et al., 2008) and this result contrasts with the lack of MSE_HF_ differences we found. To exclude that this discrepancy is due to computational aspects, we re-estimated MSE as in (Javorka et al., 2008): coarse-graining without overlapping, *m* = 2 and *r* = 0.15. This analysis too did not reveal differences between T1DM (MSE = 1.39, = 1.52, and = 1.65 at scales of 2, 3, and 4 beats respectively) and CNTR (MSE = 1.41, = 1.55, and = 1.68 at 2, 3, and 4 beats, *p* > 0.22 at each scale) groups. The shortest scales may be affected by respiration because MSE_HF_ is lower in supine volunteers breathing at high altitudes (Faini et al., 2019), where respiratory efforts are increased. Thus, different breathing patterns may have contributed to the discrepant results. In our study, the breathing rate during SUP was similar in patients (0.20 Hz) and controls (0.18 Hz). Differently from our study, the patients enrolled in (Javorka et al., 2008) had a higher body mass index than controls, a condition that might have favored deeper, more regular breathing during the one-hour long supine period. If such a respiratory variation had happened, it could have caused more predictable HRV dynamics in patients, decreasing entropy at the scales influenced by respiration as observed in (Javorka et al., 2008).

### Clinical Implications

New wearable devices make HRV easy to measure at home facilitating the early diagnosis of DCAN with HRV. This could have a major impact on daily clinical practice allowing rapid interventions and adoption of treatments after DCAN identification, like participation in rehabilitation programs based on exercise training (Bhati, Shenoy and Hussain, 2018). Caffeine supplementation could be also an option because in type-1 diabetic patients caffeine increases the HRV vagal indexes we found to be depressed in our patients (Richardson et al., 2004). ACE inhibitors might also improve the HRV vagal indexes that DCAN depresses (Kontopoulos et al., 1997). Long-term assessment of treatment effects on DCAN could be important during therapy with glucagon-like peptide 1 receptor agonists, which improve glucose metabolic parameters in type-1 diabetes (Guyton, Jeon and Brooks, 2019) but might exacerbate DCAN impairing the HRV vagal indexes (Kumarathurai et al., 2017). HRV could also help identify candidates for more stringent glycemic control with continuous glucose monitoring devices and insulin pumps (Battelino et al., 2019).

### Strengths and Limitations

A strength of our study is that the cardiovascular autonomic function is assessed through a series of autonomic maneuvers differently eliciting both cardiac and vascular responses, and by families of HRV indexes encompassing not only time and frequency domains but also more advanced complexity approaches: this allowed us to obtain detailed information on the cardiovascular autonomic control. Another strength is to consider a population of normotensive, normal-weight, mid-age patients: this allows us to reasonably exclude causes of autonomic alterations different from DCAN. However, the vagal HRV declines with age (Umetani et al., 1998). This limits the applicability of our results to older patients or children because the HRV capability to predict DCAN could differ from what we reported in our participants. Moreover, we considered the HRV methods for which the physiological correlates are better known. Complexity methods not considered in this study, like the recurrence quantification analysis (Souza et al., 2016), might provide additional information for detecting early DCAN.

### Conclusion

HRV indexes measured during a few minutes of supine rest can detect DCAN even at such an early stage where traditional autonomic tests fail to identify any abnormality. This has practical applications because HRV measures can be easily standardized and are obtainable with cheap devices in a few minutes without the active participation of the patient. Intriguingly, indexes with similar AUC have different sensitivity and specificity, suggesting that a combination of these indexes may increase the HRV predictive power.

## Data Availability

The datasets presented in this study can be found in online repositories. The names of the repository/repositories and accession number(s) can be found below: The data supporting the main findings of this study are available at doi:10.5281/zenodo.5789370 with access granted on justified request to researchers meeting the criteria for access to confidential data due to the hospital research policy and restrictions requested by the ethical committee.
