# Supplementary material for: Heart Rate Variability for the Early Detection of Cardiac Autonomic Dysfunction in Type 1 Diabetes
Source: Front Physiol. 2022 Jun 30;13:937701. doi: 10.3389/fphys.2022.937701 (PMC9281578; doi:10.3389/fphys.2022.937701)
Supplement: Supplementary file 1 [file DataSheet1.PDF]

Supplementary Material

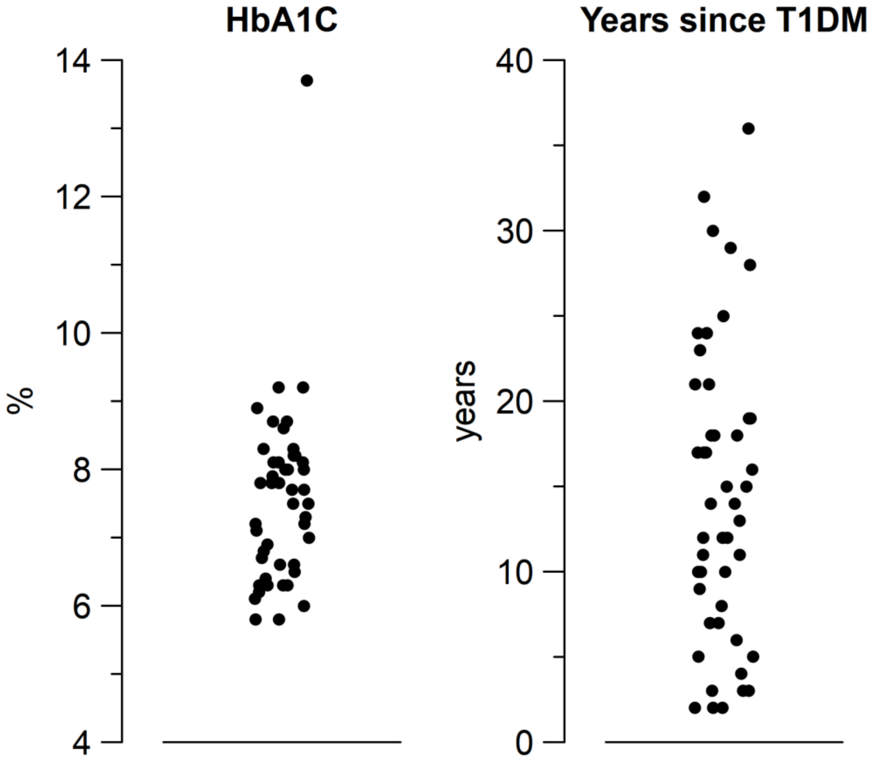

Supplementary Figure S1. Individual values of glycated hemoglobin (HbA1C) and years since the diagnosis of type-1 diabetes mellitus in the patients' group.

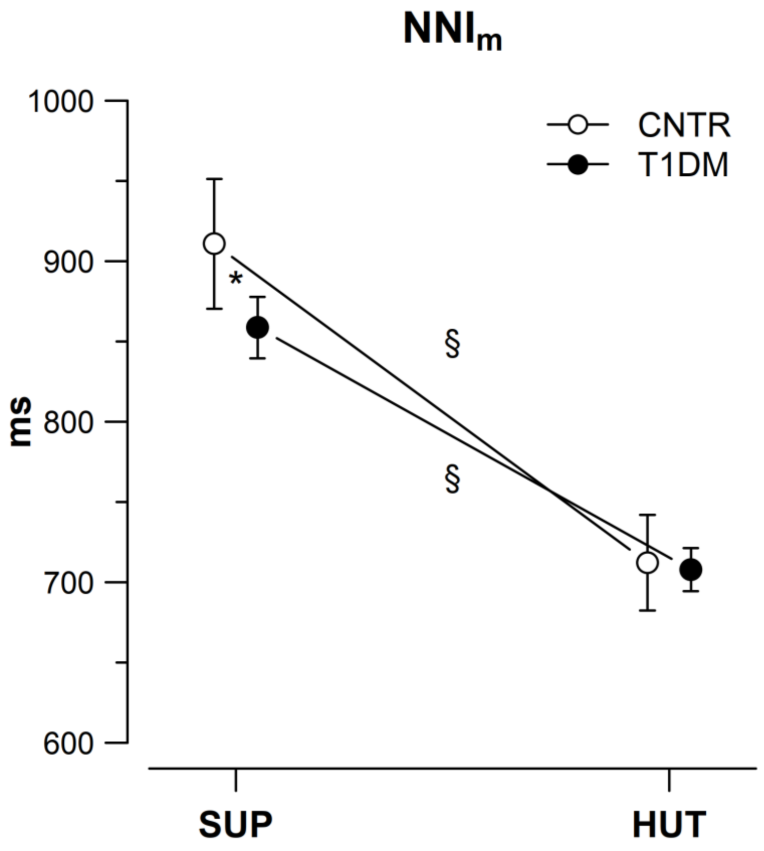

Supplementary Figure S2. Normal-to-normal interval (NNI_m) in supine (SUP) and head-up tilt (HUT) by groups. Values as median ±standard error of the median in controls (CNTR) and patients (T1DM). The * indicates differences between groups, the § differences between conditions, at the 5% significance level.

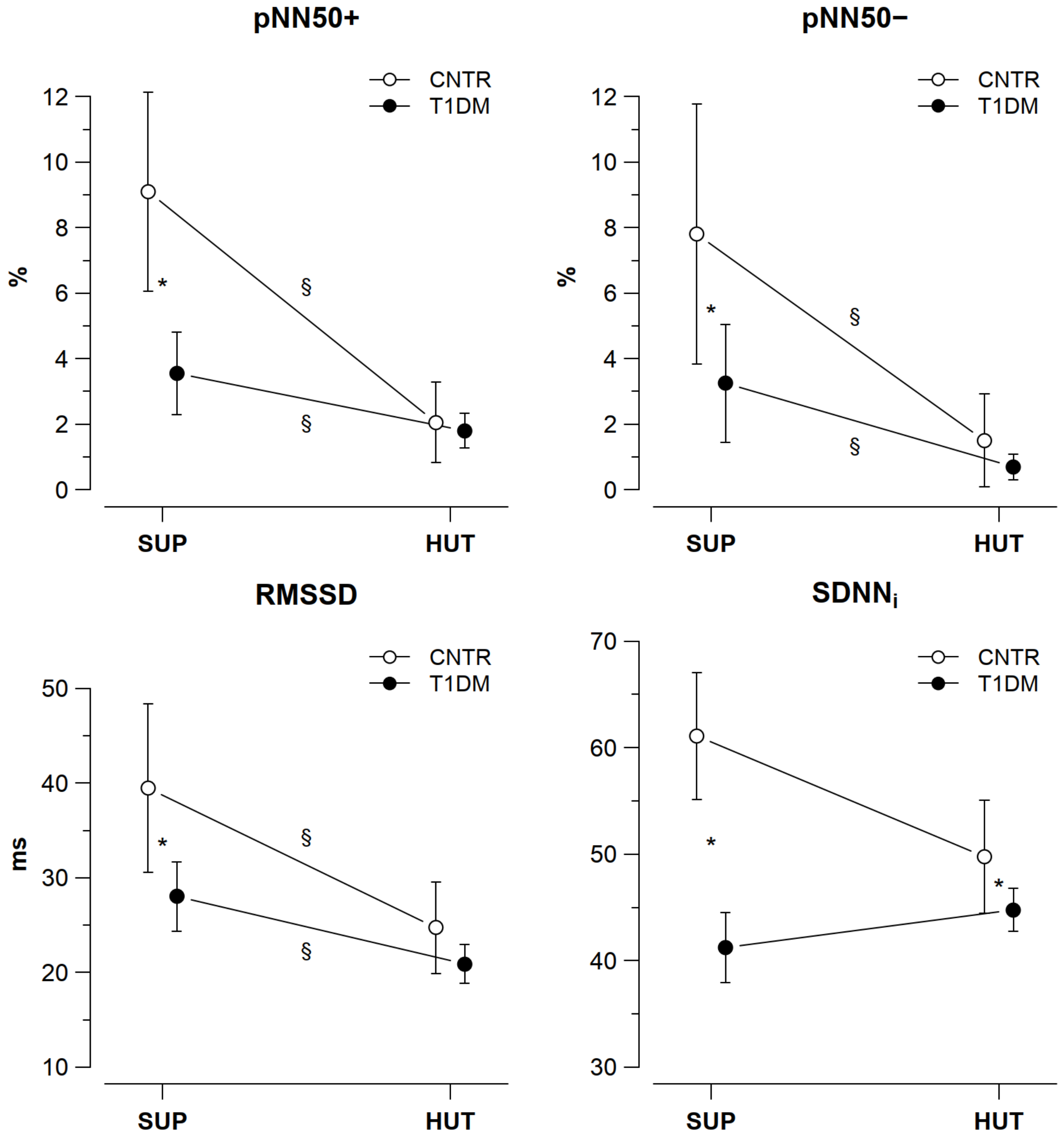

Supplementary Figure S3. Time-domain indexes of HRV in supine and head-up tilt by groups. Percentage of N-N intervals at least 50 ms longer (pNN50+) or shorter (pNN50-) than their preceding N-N interval, root mean square of successive NNI differences (RMSSD), and NNI standard deviation over a 5-min running window ($SDNN_i$): values as median ±standard error of the median. See figure S1 for symbols.

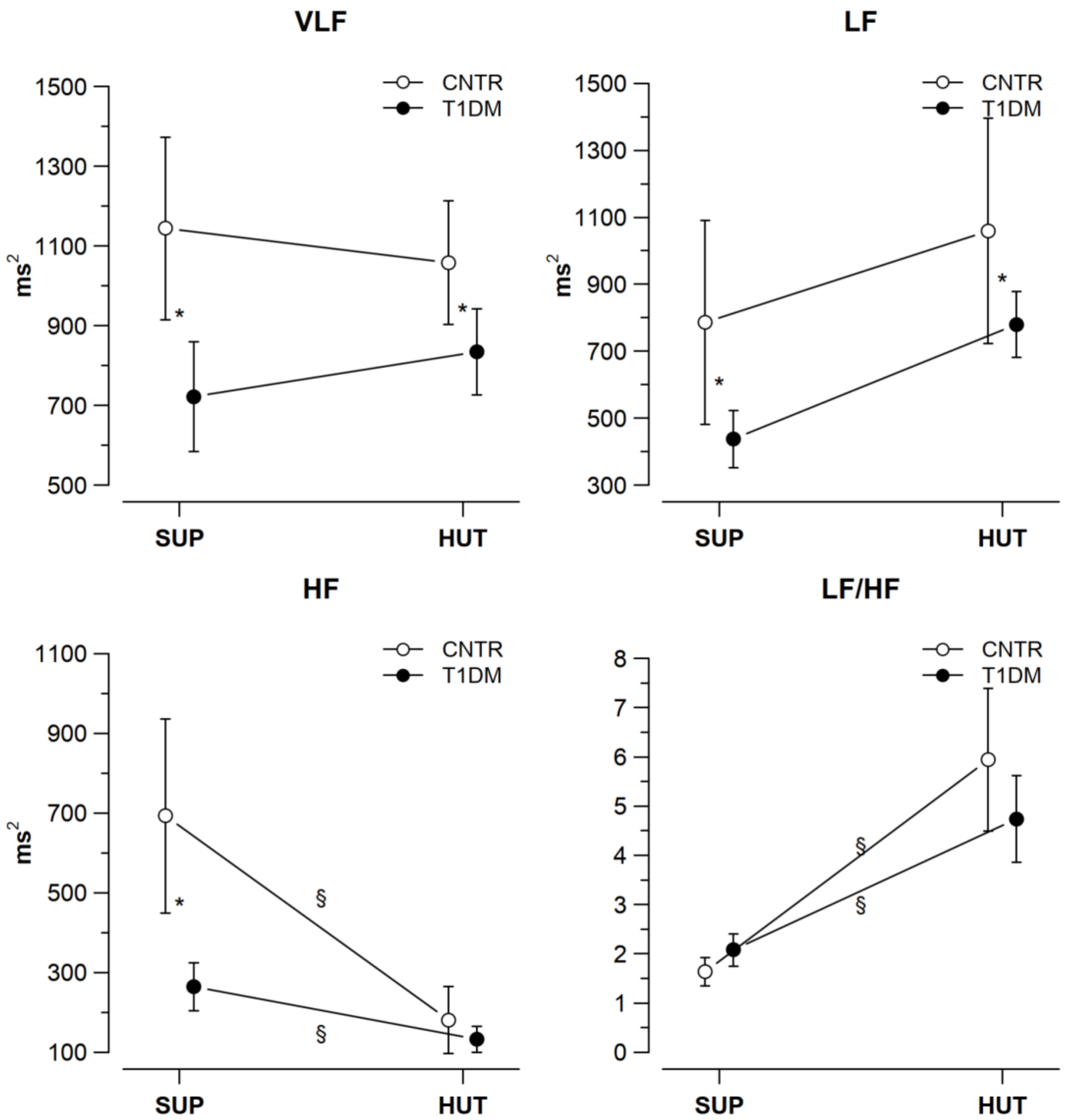

Supplementary Figure S4. Frequency-domain HRV indexes in supine and head-up tilt by groups. Spectral powers in the VLF, LF, and HF frequency bands and LF/HF powers ratio as median ±standard error of the median. See Figure S1 for symbols.

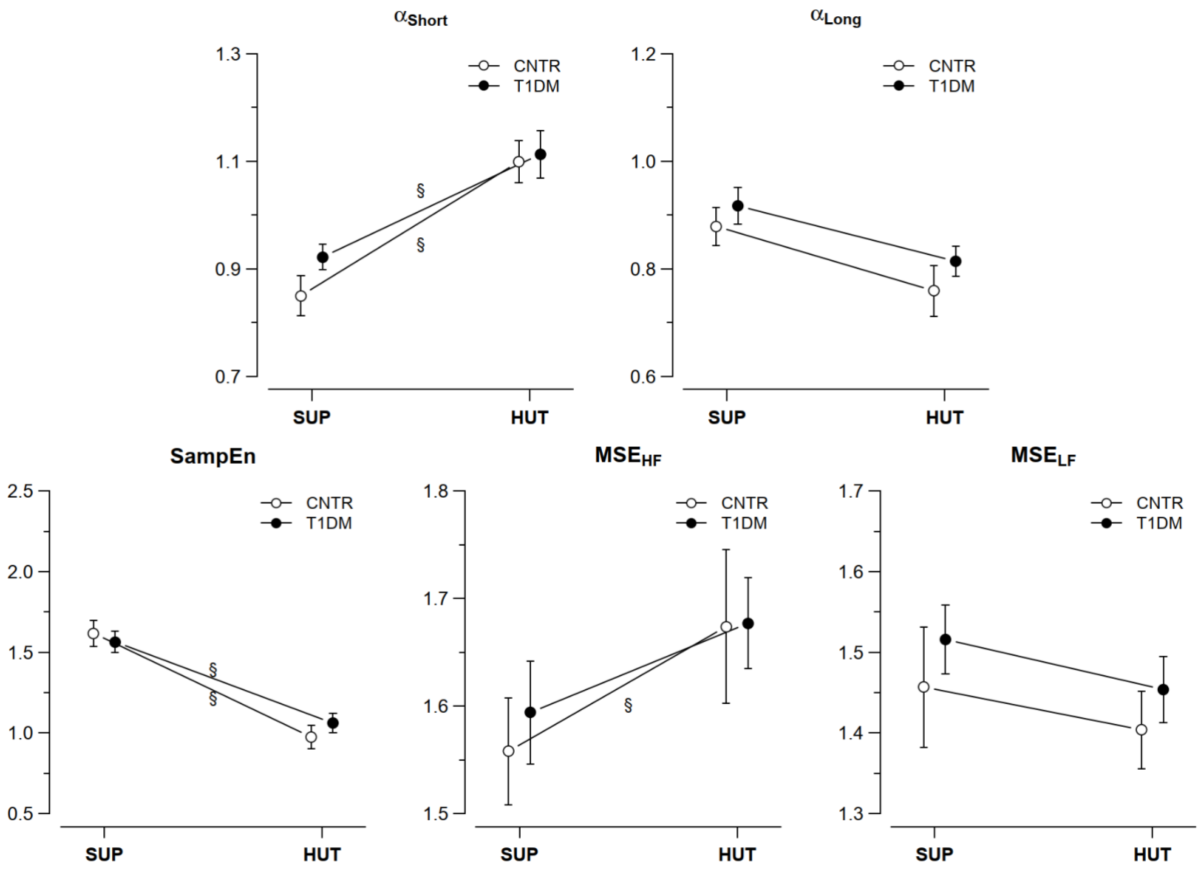

Supplementary Figure S5. Complexity-domain HRV indexes in supine and head-up tilt by groups. Upper panels: self-similarity coefficients over short-term (α_{Short}) and long-term (α_{Long}) scales; lower panels: sample entropy (SampEn) and multiscale entropy components at high (MSE_{HF}) and low (MSE_{LF}) frequencies; values as median ±standard error of the median. See figure S1 for symbols.
